# Infrared light elicits endothelium-dependent vasodilation in isolated occipital arteries of the rat via soluble guanylyl cyclase-dependent mechanisms

**DOI:** 10.3389/fphys.2023.1219998

**Published:** 2023-08-16

**Authors:** Tristan H. J. Lewis, Junqi Zhuo, Jacob X. McClellan, Paulina M. Getsy, Rita M. Ryan, Michael. J. Jenkins, Stephen J. Lewis

**Affiliations:** ^1^ Department of Neurosciences, Lerner Research Institute, Cleveland Clinic, Cleveland, OH, United States; ^2^ Department of Biomedical Engineering, Case Western Reserve University, Cleveland, OH, United States; ^3^ Department of Pediatrics, Case Western Reserve University, Cleveland, OH, United States; ^4^ Departments of Pharmacology, Case Western Reserve University, Cleveland, OH, United States; ^5^ Functional Electrical Stimulation Center, Case Western Reserve University, Cleveland, OH, United States

**Keywords:** infrared light, occipital arteries, endothelium-dependent vasodilation, nitric oxide, soluble guanylate cyclase

## Abstract

The left and right occipital arteries provide blood supply to afferent cell bodies in the ipsilateral nodose and petrosal ganglia. This supply is free of an effective blood-ganglion barrier, so changes in occipital artery blood flow directly affect the access of circulating factors to the afferent cell bodies. The application of infrared (IR) light to modulate neural and other cell processes has yielded information about basic biological processes within tissues and is gaining traction as a potential therapy for a variety of disease processes. To address whether IR can directly modulate vascular function, we performed wire myography studies to determine the actions of IR on occipital arteries isolated from male Sprague-Dawley rats. Based on our previous research that functionally-important differences exist between occipital artery segments close to their origin at the external carotid artery (ECA) and those closer to the nodose ganglion, the occipital arteries were dissected into two segments, one closer to the ECA and the other closer to the nodose ganglion. Segments were constricted with 5-hydroxytryptamine to a level equal to 50% of the maximal response generated by the application of a high (80 mM) concentration of K^+^ ions. The direct application of pulsed IR (1,460 nm) for 5 s produced a rapid vasodilation in occipital arteries that was significantly more pronounced in segments closest to the ECA, although the ECA itself was minimally responsive. The vasodilation remained for a substantial time (at least 120 s) after cessation of IR application. The vasodilation during and following cessation of the IR application was markedly diminished in occipital arteries denuded of the endothelium. In addition, the vasodilation elicited by IR in endothelium-intact occipital arteries was substantially reduced in the presence of a selective inhibitor of the nitric oxide-sensitive guanylate cyclase, 1H-[1,2,4]oxadiazolo [4,3-a]quinoxalin-1-one (ODQ). It appears that IR causes endothelium-dependent, nitric-oxide-mediated vasodilation in the occipital arteries of the rat. The ability of IR to generate rapid and sustained vasodilation may provide new therapeutic approaches for restoring or improving blood flow to targeted tissues.

## 1 Introduction

The left and right nodose ganglia house the cell bodies of ipsilateral vagal afferent fibers whereas the left and right petrosal ganglia house the cell bodies of ipsilateral glossopharyngeal afferent fibers (Abboud et al., 1976; Thorén, 1979; Spyer, 1981; Lacolley et al., 2006a; Lacolley et al., 2006b; Iturriaga et al., 2007; Retamal et al., 2014). The arterial blood supply to ganglia is of well-established importance to the activity of sensory cell bodies, as is whether these blood supplies have true blood-ganglion barriers that allow for the exchange of nutrients such as oxygen and glucose but prevent larger molecular weight factors to access the sensory cell bodies (see Davis and Story, 1943; Chungcharoen et al., 1952; Chai and Wang, 1966; Chai et al., 1967; Jacobs and Comroe, 1971; Pugh and Kalia, 1982; van der Krans and Hoogland, 1983; Ling and Wong, 1988; Ten Tusscher et al., 1989; Lacolley et al., 2006a; Lacolley et al., 2006b). Early evidence for the lack of an effective blood-ganglion barrier in the nodose ganglion was elegantly demonstrated by Ten Tusscher et al. (1989). They found that intravenous injections of large molecular weight horseradish peroxidase in rats resulted in its ready appearance between sensory ganglion cells and their satellite cells in the nodose ganglion, whereas it was absent in the interstitial space between ganglion cells and their satellite cells within superior cervical, medial cervical and pterygopalatine ganglia (although horseradish peroxidase lined satellite cell membranes).

There is now compelling evidence that the nodose and petrosal ganglia are supplied by a branch of the internal carotid artery, which has a tight blood-ganglion barrier that denies blood-borne factors (other than nutrients) access to sensory cell bodies (Ten Tusscher et al., 1989; Lacolley et al., 2006a; Lacolley et al., 2006b). Intriguingly, the nodose and petrosal ganglia also receive direct arterial blood supply from their ipsilateral occipital arteries, which branch off respective external carotid arteries (ECA). Both ganglia, despite the presence of satellite cells, are devoid of effective blood-ganglion barriers such that circulating factors like 5-hydroxytryptamine (5-HT) gain ready access to sensory cell bodies in the ganglia to affect the activity of vagal and glossopharyngeal afferents (Ten Tusscher et al., 1989; Lacolley et al., 2006a; Lacolley et al., 2006b). We postulated that circulating factors such as 5-HT, angiotensin II, arginine vasopressin, and S-nitrosothiols in the occipital arterial blood generated by cardiorespiratory challenges (e.g., hypertension, obstructive sleep apnea, diabetes, sepsis) may directly modulate vagal and glossopharyngeal afferent activity via actions on their functionally-active membrane-bound receptors that exist on the plasma membranes of the sensory cell bodies (Higashi and Nishi, 1982; Lewis et al., 1986; Allen et al., 1988; Phillips et al., 1990; Gao et al., 1992; Widdop et al., 1992; Owen at el, 2005; Lacolley et al., 2006c; Lewis et al., 2006; Moreira et al., 2009).

We have examined the *in vitro* responsiveness of rat occipital arteries via wire myography methods (Chelko et al., 2013; Chelko et al., 2014; Lewis et al., 2021) and found significant differences in responsiveness to a variety of agonists based on the proximity of the arterial segment to its origin at the external carotid artery (ECA). When the occipital arteries were bisected and examined, we found that the distal segment (closer to the nodose ganglion) was much more reactive than the proximal segment (that directly arising from the ECA) to agonists including 5-HT, a selective 5-HT_2_ receptor agonist, α-CH_3_-5-HT, arginine vasopressin (AVP), and a selective V1 receptor agonist, Phe^2^, Ile^3^, and Orn^8^-vasopressin (Chelko et al., 2013; Chelko et al., 2014). More recently, we determined that the segmental differences in vasoreactivity were largely dependent on the presence of an intact endothelium (Lewis et al., 2021). When the endothelium was removed, though both segments were more reactive to the same agonists, the increase in the reactivity of the proximal segment meant the total responses were almost equal to the distal (Lewis et al., 2021).

Direct application of IR light has been demonstrated as a method of optical stimulation of nerves that does not require additional interventions like the addition of photoreactive molecules or genetic manipulation (see Thompson et al., 2014 for review). The direct application of IR light to neural tissues can elicit a spatially selective inhibition (Duke et al., 2013; Lothet et al., 2017), and the extracellular ion concentration can modulate the sensitivity of neural tissues to IR light (Zhuo et al., 2021). There is compelling evidence that the application of red/near IR elicits endothelium-dependent vasodilation in murine facial arteries (Keszler et al., 2017) and hindlimb (Keszler et al., 2022), and that this vasodilation may involve the release of nitric oxide from pre-formed pools of S-nitrosothiols and/or nitrosylated proteins (Keszler et al., 2018; Keszler et al., 2019; Weihrauch et al., 2021). Based on the evidence in other arteries, it may be possible that these pre-formed pools of S-nitrosothiols in murine facial arteries are stored within cytoplasmic vesicles that are subject to exocytosis (Seckler et al., 2020). However, the mechanisms by which pulsed IR (between the wavelength of 1,400–2,100 nm) are thought cause neuromodulation involves the generation of temperature changes to modulate action potentials (Ganguly et al., 2019), whereas light in the 670 nm range affects vasoreactivity by liberating vasoactive nitric oxide precursor species via photobiomodulation (Pierrefiche et al., 2007; Ganguly et al., 2019). So, the mechanisms by which IR elicits physiological responses have been shown to be substantively different based on the specific wavelengths used (600 range versus 1,400–2,100). As members of our group and others have demonstrated that IR light in the 1,460 range has specific neuromodulatory effects (Duke et al., 2013; Lothet et al., 2017; Ganguly et al., 2019; Zhuo et al., 2021) not seen at other wavelengths, we wanted to test IR in this range directly on vascular function, specifically in a vessel type with a demonstrated difference in endothelial function, but consistent smooth muscle function, along the length of the vessel (Lewis et al., 2021). We hypothesized that IR light at 1,460 nm would have rapid vasoactive effects that are largely dependent on the presence of a functional endothelium. At present, there is no published evidence as to the potential effects of IR in the 1,460 nm range on vascular tone or endothelial function.

Based on the importance of the occipital arterial blood supply to the function of sensory cell bodies within the nodose ganglion, the major aims of the present study were to 1) determined the effects of brief, pulsed episodes of IR (using a 1,460 nm laser diode) on the vasoreactivity of the proximal (those closer to the ECA) and distal (those closer to the nodose ganglion) occipital artery segments from (presumably normotensive) adult male Sprague-Dawley rats, 2) to determine the role of the vascular endothelium in the responses of the proximal and distal segments to IR, and 3) establish whether the selective inhibitor of nitric oxide-mediated activation of soluble-guanylate cyclase, 1H-[1,2,4]oxadiazolo [4,3-a]quinoxalin-1-one (ODQ) (43), modulates IR responses in intact arteries.

## 2 Materials and methods

### 2.1 Permissions, rats, and surgical procedures

All studies were carried out in accordance with the NIH Guide for Care and Use of Laboratory Animals (8th edition, revised in 2011) and in strict compliance with the ARRIVE (Animal Research: Reporting of *In Vivo* Experiments) guidelines. All protocols involving the use of rats were approved by the Animal Care and Use Committee of Case Western Reserve University. Adult male Sprague Dawley rats (300–350 g body weight) were purchased from *Harlan Industries* (Madison, WI, United States). The rats were given 4 days to recover from transport before use. All studies were done in a quiet room with a relative humidity of 51% ± 2% at a room temperature of 21.2°C ± 0.2°C.

### 2.2 Artery isolation and small vessel myography setup

Rats were decapitated and their heads were immediately placed on ice in a physiological saline solution (PSS) containing (mM) NaCl 118, NaHCO_3_ 24, KCl 4, glucose 5.9, MgSO_4_ 1, NaH_2_PO_4_ 0.435, and CaCl_2_ 1.8. Occipital arteries (OA, 250–400 μm internal diameter) were isolated and bisected into proximal (closer to ECA) and distal (closer to nodose ganglion) tubular segments and mounted separately on small vessel myographs (Model 500A, *Danish Myo Technology*, Denmark), as detailed previously (Chelko et al., 2013; Chelko et al., 2014; Lewis et al., 2021). After equilibrating for 30 min in PSS gassed with 21% O_2_, 5% CO_2_, and 74% N_2_ (pH 7.4, 37.0°C), occipital arteries were stretched to a force calculated as previously described (Mulvany and Halpern, 1977) for arteries that experience systemic blood pressure. Arteries were normalized by passively stretching in 2 mN increments every 60 s until an internal circumference (IC) was reached that equated to a transmural pressure of 13.3 kPa (IC_100_). Next, the IC_100_ is multiplied by the normalization factor of 0.9 to determine the optimal IC for each segment to produce maximal vascular reactivity (IC_1_), and the IC is adjusted to this calculated value. Maximal contractile responses of occipital artery segments to a depolarizing stimulus were established by (HiK) depolarization, i.e., exposing them to potassium PSS with 80 mM K^+^ (isotonic replacement of Na^+^ by K^+^) as detailed previously (Robertson et al., 2003; Robertson et al., 2007; Robertson et al., 2008; Chelko et al., 2013; Chelko et al., 2014).

### 2.3 Removal of the endothelium

All vessels were given one exposure to HiK for 2 min. After washing with PSS, endothelium denudation was accomplished by the gentle rubbing of the luminal surface of IPA with a human forearm hair. To ensure that this procedure had not damaged the smooth muscle, we exposed denuded OA segments to HiK. Segments that did not generate at least 75% of the tension measured during the response to 5HT of the equilibration procedure were excluded. Endothelial disruption was confirmed at the end of the experiment by the abolition of the vasodilator response to acetylcholine (1 μM) in arteries preconstricted with 5HT (10 μM).

### 2.4 Evaluation of the effects of IR light on vascular reactivity

**FIGURE 1:**
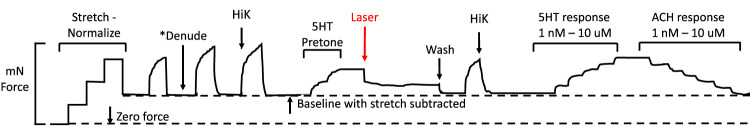
Representation of a standard myography experiment evaluating the effects of IR light. First, the vessel is stretched via the automated normalization process to a tension equivalent to the appropriate mean internal pressure. Second, one HiK exposure is conducted. If required, the endothelium is then removed, followed by two more HiK exposures. Next, 5-hydroxytryptamine (5-HT pre-tone) is added to each segment to elicit approximately 50% of the maximal HiK response. The IR light fiber is positioned over the artery, then the segment is exposed to the IR. Next, the 5HT is washed from the bath, and another HiK is performed. Finally, concentration-response curves are established for 5HT and acetylcholine (ACH).

A representative diagram of the IR experiments is provided in Figure 1. Vessels were pre-constricted with 5HT to a level equivalent to 50% of the maximal response generated by HiK depolarization. Next, a 400 μm diameter optic fiber (P400 VIS-NIR, *Ocean Optics*, Largo, FL) was positioned 30 μm from optic fiber tip to tissue using a micromanipulator. A 1460 nm laser diode (MCM-102, *SemiNex*, Peabody, MA) delivered 200 μsec pulses of 0.3 mJ IR at 200 Hz for a 5-s duration. The fiber-to-artery distance was achieved by using a micromanipulator to lower the fiber into contact with the artery and then retract by 30 µm. As the arteries are stretched between the wires, the fiber was positioned perpendicular to the plane of the vessel, applying light radially to the tissue. For certain vessels, 10 μM ODQ was added into the PSS at least 10 min before IR light application.

### 2.5 Radiant exposure calculations

Radiant exposures (radiant energy received by a surface per unit area, J/cm^2^) at the tip of the optical fiber were calculated by dividing the pulse energies by the laser spot size which is the area of the fiber tip (Jenkins et al., 2010). Pulse energies were measured using a pyroelectric energy meter (Nova II, Ophir Photonics), and spot size using the average fiber diameter. Laser light was delivered via a 400 ± 8 µm diameter flat-polished multi-mode optical fiber (*Ocean Optics*, Largo, FL). We, therefore, calculated radiant exposure to be:

$$\left(pulse\;energy\;in\;mJ\right)/\left(\pi *{\left(radius\;of\;fiber\;tip\;in\;um\right)}^{2}\right)=radiant\;exposure\;in\;J/{cm}^{2}$$

$$\left(0.3\;mJ\right)/\left(\pi *{\left(200\;um\right)}^{2}\right)=0.239J/{cm}^{2}\left(at\;fiber\;tip,per\;pulse\right)$$

### 2.6 Effects of IR light on agonist-induced vasoconstriction and vasorelaxation

Concentration-dependent response curves were established for 5-HT (1 nM–10 μM) and then acetylcholine (ACh, 1 nM to 10 μM) on occipital arteries and internal carotid arteries after exposure to the IR light. 5-HT and ACh responses were also collected from separate occipital artery segments that were not exposed to IR to provide naïve agonist responses to determine the IR effect on the tissue response. ACh responses were tested using the maximal constriction generated by 10 μM 5-HT.

### 2.7 Data analyses

All data are presented as mean ± SEM. Data for IR responses (Figures 3–5) were evaluated using repeated measures two-way ANOVA with the Geisser-Greenhouse correction. Data in Figures 3A, B were further compared via Šídák’s multiple comparisons test. Data comparing pre-constriction levels, responses to HiK before and after IR (Figure 6), and data describing the cumulative addition of 5-HT (Figure 7) and ACH (Figure 8) were analyzed via ordinary one-way ANOVA with Tukey’s multiple comparisons test. Statistical analyses were performed using GraphPad Prism software (GraphPad Software, Inc., La Jolla, CA). Each n value refers to a biological replicate.

## 3 Results

### 3.1 Effects of IR light on occipital artery segments with 5HT-induced pretone

**FIGURE 2:**
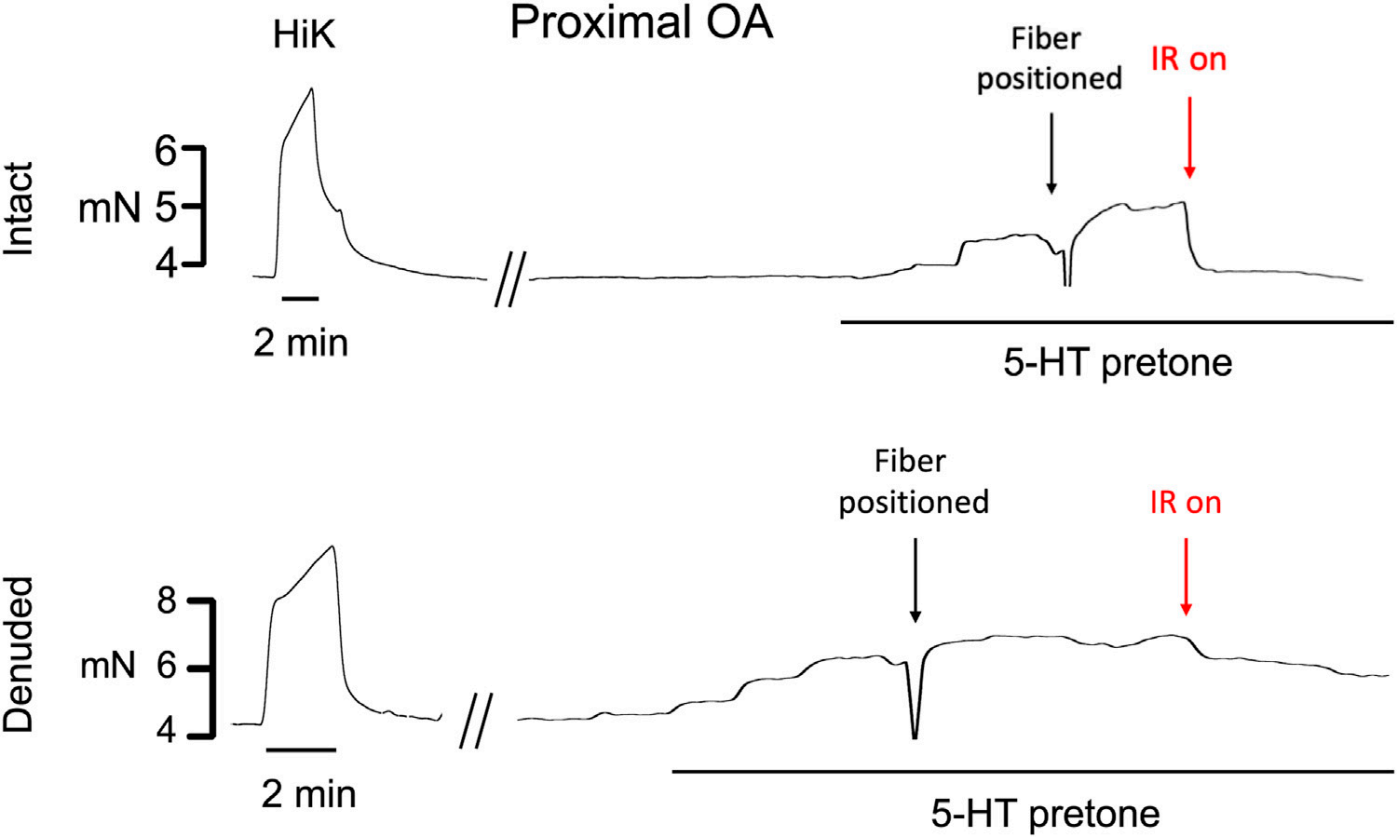
Typical examples of the effects of a 5-s exposure of infrared light to the contractile force (mN) of intact and endothelium-denuded proximal occipital artery segments. The term “HiK” and the time bar below the trace denote the application of 80 mM K^+^ PSS for 2 min 5-hydroxytryptamine (5-HT pre-tone) was added to each segment to elicit approximately 50% of the maximal HiK response. The term “Fiber Positioned” refers to the use of a micromanipulator to touch the fiber to the transducer arm which registers as a lowering of force, retracting 30 μm, then using the pilot light to position over the artery center. IR light is then delivered 200 μs pulses of IR at 200 Hz for a 5-s duration.

**FIGURE 3:**
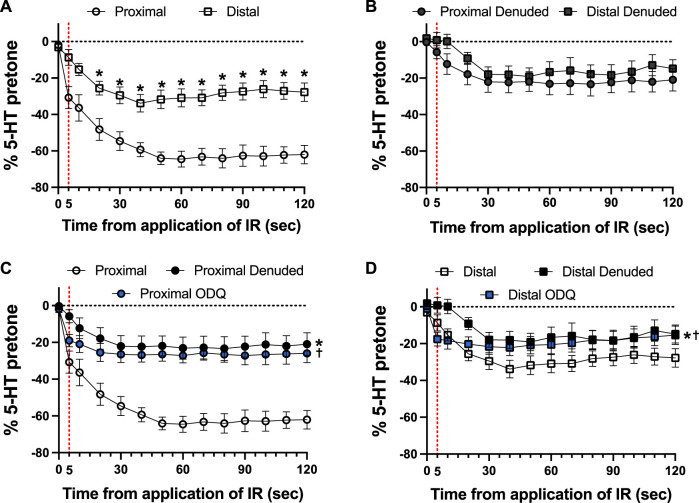
Responses to 5-s IR light exposure of intact and endothelium-denuded proximal and distal occipital artery segments with and without 10 μM ODQ. The data are presented as mean ± SEM of the responses expressed as a percentage of agonist-induced constriction (% 5-HT pre-tone). *N* = 12 for intact arteries, *N* = 7 for endothelium-denuded, and *N* = 6 ODQ. Data are compared via repeated measures two-way ANOVA with the Geisser-Greenhouse correction. **(A, B)**: *p < 0.05 between Distal and Proximal at each timepoint via Šídák’s multiple comparisons test. **(C, D)**: *p < 0.05 between Denuded and Intact, ^†^ *p* < 0.05 between ODQ and Intact, and ^‡^ *p* < 0.05 between ODQ and Denuded.

Typical examples of the effects of a 5-s IR exposure on the contractile force of intact and endothelium-denuded proximal occipital artery segments are shown in Figure 2. The IR-induced responses were substantially greater in the intact segments (top panel) than in the endothelium-denuded segments (bottom panel). The mean and standard error of the pre-constriction values, as calculated as a percentage of the maximal HiK^+^ response, are as follows: Proximal = 39.9 ± 5.4 (*n* = 12), Proximal Denuded = 51.4 ± 7.6 (*n* = 7), Proximal ODQ = 48.4 ± 8.8 (*n* = 6), Distal = 54.7 ± 4.4 (*n* = 12), Distal Denuded = 57.9 ± 6.3 (*n* = 7), and Distal ODQ = 58.8 ± 9.4 (*n* = 6). These pre-constriction values were not determined to be significantly different via ordinary one-way ANOVA. Vasodilation caused by IR was significantly greater in the intact proximal OA segments versus the intact distal segments (Figure 3A) and was sustained for at least 2 min after termination of the IR exposure. While there was a small, yet significant, reduction in the response in the distal occipital artery segment after the removal of the endothelium (Figure 3D), the effects of denudation on the IR response were much more pronounced in the proximal arteries (Figure 3C). With the removal of the endothelium, both the proximal and distal occipital artery segments had a small response to IR, but the responses were not distinguishably different (Figure 3B). The addition of 10 μM ODQ also significantly reduced the response to IR light, particularly in the proximal endothelium-intact segments.

**FIGURE 4:**
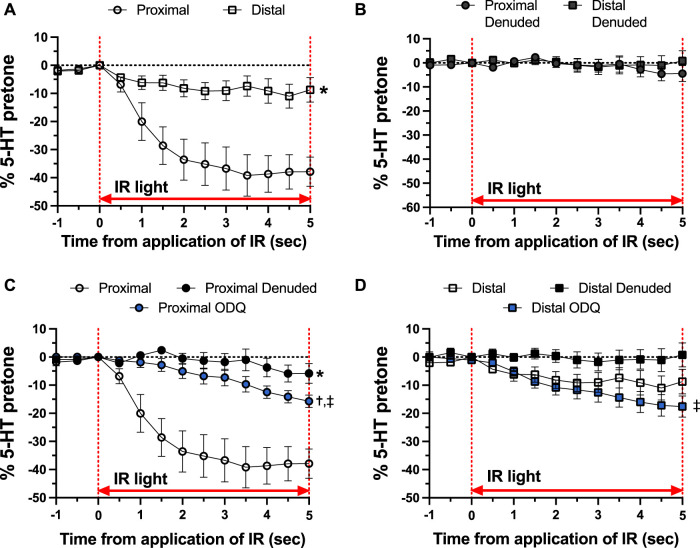
Responses during 5-s IR light exposure in intact and endothelium-denuded proximal and distal occipital artery segments with 10 μM ODQ. The data are presented as mean ± SEM of the responses expressed as a percentage of agonist-induced constriction (% 5-HT pre-tone). *N* = 12 for intact arteries, *N* = 7 for endothelium-denuded, and *N* = 6 ODQ. Data are compared via repeated measures two-way ANOVA with the Geisser-Greenhouse correction. **(A, B)**: *p < 0.05 between Distal and Proximal **(C, D)**: *p < 0.05 between Denuded and Intact, ^†^ *p* < 0.05 between ODQ and Intact, and ^‡^ *p* < 0.05 between ODQ and Denuded.

**FIGURE 5:**
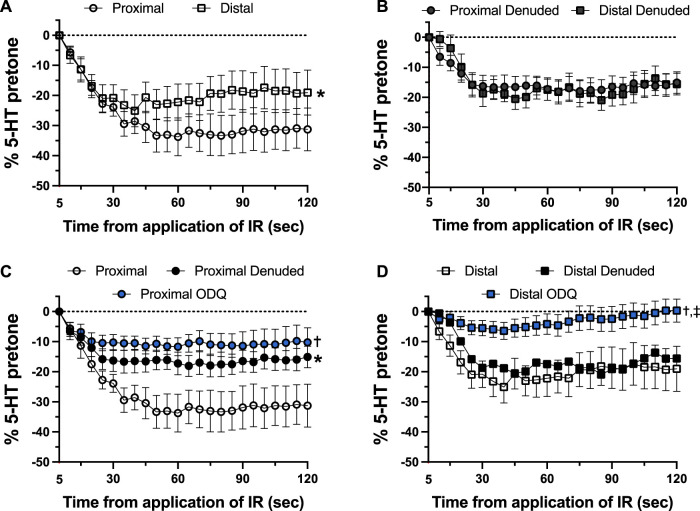
Changes in tension immediately after 5-s IR light exposure of intact and endothelium-denuded proximal and distal occipital artery segments with 10 μM ODQ. The responses during 5-s IR light exposure have been subtracted, and all values at the moment the IR light was turned off are set as 0. The data are presented as mean ± SEM of the responses expressed as a percentage of agonist-induced constriction (% 5-HT pre-tone). *N* = 12 for intact arteries, *N* = 7 for endothelium-denuded, and *N* = 6 ODQ. Data are compared via repeated measures two-way ANOVA with the Geisser-Greenhouse correction. **(A, B)**: *p < 0.05 between Distal and Proximal **(C, D)**: *p < 0.05 between Denuded and Intact, ^†^ *p* < 0.05 between ODQ and Intact, and ^‡^ *p* < 0.05 between ODQ and Denuded.

As shown in Figure 4, there was a distinct difference in the responses of the occipital artery segments during the 5-s exposure to IR. The endothelium-intact proximal segments responded substantially more than the distal segments (Figure 4A). In contrast, the vascular tone of both segments was virtually unchanged during IR exposure when the endothelium was removed (Figure 4B). The addition of 10 μM ODQ significantly reduced the response to IR, but only in the proximal segments (Figures 4C, D). In both segments, ODQ was not as effective as denudation at blocking the IR response during exposure to IR. In addition, there was a vasodilator response that was initiated once the IR was turned off. This effect is demonstrated in Figure 5, where the responses during the 5 s of IR exposure were subtracted, and what is displayed represents the responses only after the IR was turned off. The response was greater in the intact proximal versus distal arteries (Figure 5A). The responses of both denuded segments were again indistinguishable (Figure 5B). Furthermore, While 10 μM ODQ did diminish the response in the proximal OA similar to that of denudation (Figure 5C), the effect was far more pronounced in the distal segments (Figure 5D).

### 3.2 K^+^- and agonist-induced vasoreactivity of occipital artery segments before and after IR

**FIGURE 6:**
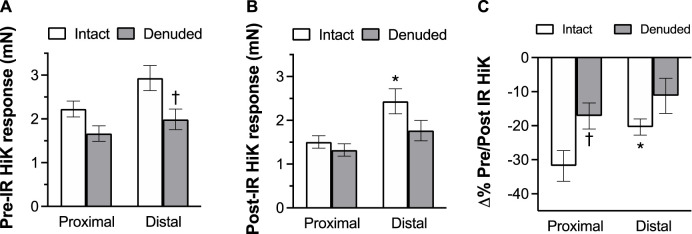
The effects of IR exposure on the response to the changes in tension (mN) elicited by the addition of 80 mM K + PSS (HiK) in proximal and distal intact, and endothelium-denuded OA segments. HiKs were conducted before **(A)** and after **(B)** exposure to 5 s of IR light. Percent changes in the responses are shown in the **(C)**. The data are presented as mean ± SEM. *N* = 12 for intact arteries, *N* = 7 for endothelium-denuded. *p < 0.05 indicates a significant difference between proximal and distal, ^†^ *p* < 0.05 between intact and denuded.

**FIGURE 7:**
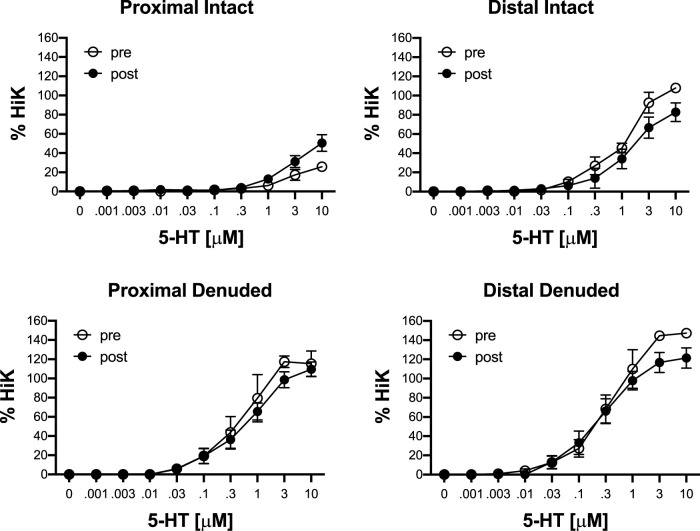
Responses to cumulative addition of 5-hydroxytryptamine (5-HT, 0.001–10 μM) before (pre) and after (post) 5-s IR light exposure of intact and endothelium-denuded proximal and distal occipital artery segments. The data are presented as mean ± SEM as a percentage of the response to 80 mM K^+^ (HiK). *N* = 6 for all. *p < 0.05 indicates a significant difference between pre and post.

**FIGURE 8:**
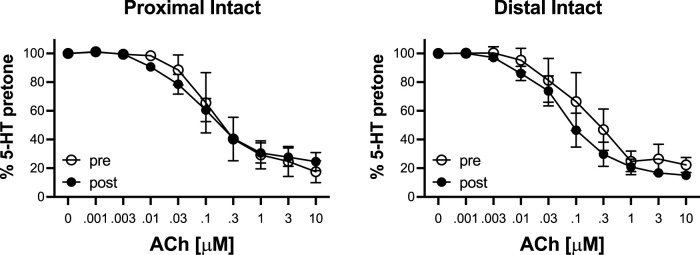
Responses to cumulative addition of acetylcholine (ACh, 0.001–10 μM) before (pre) and after (post) 5-s IR light exposure of intact and proximal and distal occipital artery segments. The data are presented as mean ± SEM as a percentage of the response to 80 mM K^+^ (HiK). *N* = 4 for “pre” and *N* = 6 for “post” groups. *p < 0.05 indicates a significant difference between pre and post.

To determine the effects of IR on vasoreactivity beyond the actual vasodilator response shown above, HiKs were performed after the IR exposures and after the 5-HT had been washed from the bath. The segments were allowed at least 20 min to return to baseline (Figure 6). Increases in tension from the third HiK depolarization conducted during the setup procedure (left panel) and after the IR experiment (middle panel) are compared and shown as a percent change (right panel). The intact and denuded proximal occipital arteries displayed a 32% and 17% decrease in HiK response, respectively. The intact and denuded distal HiK response decreased by 20% and 11%, respectively. To evaluate any effect of IR on agonist-induced vasoconstriction, a concentration-response to 5-HT was determined before and after the application of IR in a separate group of occipital artery segments (Figure 7). Unlike the changes in the HiK responses, there was no substantial difference in the responses to 5-HT observed before and after IR. Similarly, and perhaps most importantly, no difference was observed in the magnitude of the endothelium-dependent vasodilation elicited by ACh before and after IR in the intact occipital artery segments (Figure 8).

## 4 Discussion

The present study demonstrates that a brief (5 s) application of light in the infrared spectrum (1,460 nm) elicits a rapid and sustained vasodilator response in isolated rat occipital arteries. The key findings were that 1) IR elicited a far greater vasorelaxant response in the proximal occipital artery segment (closer to the ECA) than the distal occipital artery segments (closer to the nodose ganglion), 2) IR-induced vasorelaxations were largely dependent on the presence of intact endothelium, 3) the vasorelaxations were markedly diminished in the presence of the soluble cyclase inhibitor, ODQ, and 4) the vasorelaxations were sustained well after cessation of the IR exposure. Our previous study comparing the reactivity of proximal and distal occipital artery segments showed that endothelium-dependent vasodilators generate greater relaxant responses in the proximal occipital artery segment than in the distal segment whereas the relaxant responses elicited by the nitric oxide donor, MAHMA NONOate, were similar in both segments (Lewis et al., 2021). As such, it is tempting to assume that the greater vasorelaxant effect of IR on proximal arteries is due to unique properties (e.g., structural proteins, signaling pathways, subcellular organelles) not shared by the distal arteries.

The endothelium is a responsive and heterogenous network of functional cells (Chistiakov et al., 2017). The segmental differences in endothelial function between the proximal and distal segments most likely result from physiological necessity related to the position of the proximal segment to the common carotid artery. Factors driving the heterogeneity of endothelial cells include shear stresses induced by laminar versus turbulent flow over the surface of the cells via mechanosensitive signaling pathways (Chistiakov et al., 2017). Shear stress induces differentiation of endothelial cells (Wang et al., 2005), impacts the morphology of the cells including expression of organelles (Potter et al., 2011), and counteracts dysfunction of endothelial cells caused by static blood flow conditions (Lee et al., 2017). The emergence of the occipital at a near-right angle from the external carotid artery raises the likelihood of turbulent blood flow in the segment proximal to the external carotid artery (see Figure 1 of Lewis et al., 2021) that becomes increasingly laminar as blood cells enter the more distal segments. This difference in laminar/non-laminar flow over the length of the occipital artery could significantly impact the phenotype, and therefore the function of the vascular endothelium. It is also possible that the unique functionality of the proximal segment may involve the processes that allow this segment to establish a critical closing pressure, the arterial blood pressure below which arteries are closed or conversely the arterial blood pressure that elicits opening of a closed artery (López-Magaña et al., 2009; Moraes et al., 2021; Liang et al., 2022), whereas the distal segment close to the nodose ganglion has no such role to play.

IR light is readily absorbed by water, converting to thermal energy and therefore the effects of IR light on vasodilation could be regulated by a heat-sensitive mechanism. Absorption of IR light by water molecules produces a rapid thermal transient increase that can depolarize target cells (Shapiro et al., 2012), and cause direct neural activation (Wells et al., 2007), but changes in current across membranes are driven by the rate of change of temperature (dT/dt heat shock), not overall changes in temperature (Liu et al., 2014), suggesting that the brief targeted IR light application in this study could be sufficient to elicit the responses we report. Potential targets of this thermal energy are thermosensitive ion channels such as transient receptor potential (TRP) channels, which are known to play important roles in both endothelium-dependent vasodilation and in the vascular smooth muscle cells themselves (see Earley and Brayden, 2015 for review). While there is evidence that activation of TRP channels elicits in the endothelium elicits vasodilation, these mechanisms appear to mostly be independent of NO (Zhang and Gutterman, 2011), which does not fully explain our data given the great reduction in response in the presence of ODQ. We have provided evidence that hyperthermia modulates vascular reactivity and baroreflex function in conscious rats (Massett et al., 2000) and that the hemodynamic adjustments to heat stress in the rat involve the release of neurogenic/endothelial nitrosyl factors (Kregel et al., 1997). We have also provided *in vitro* evidence that raising the temperature of bath solutions to hyperthermic levels alters resting vascular tone and responsiveness to vasodilator and vasoconstrictor compounds (Massett et al., 1998a; Massett et al. 1998b; Massett et al., 1999). The role of heat-sensitive mechanisms may at least explain the consistent ≈20% vasodilation that occurred regardless of the segment, and in the absence of the endothelium or presence of ODQ.

**FIGURE 9:**
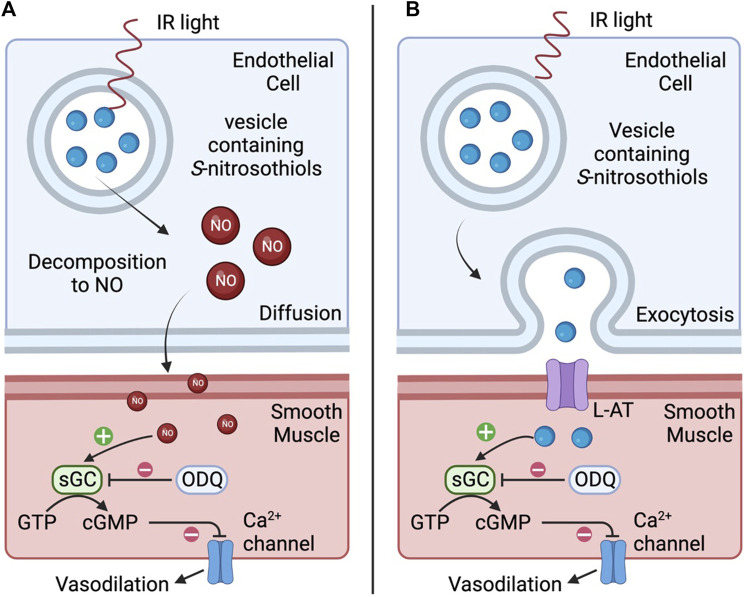
Representative diagram of two potential mechanisms by which infrared light (IR) elicits vasodilation. In **(A)**, IR directly degrades preformed S-nitrosothiols to nitric oxide (NO) which diffuses across the membrane and relaxes adjacent vascular smooth muscle via soluble-guanylate cyclase (sGC)/cGMP-dependent mechanisms. In **(B)**, IR may stimulate vesicles to fuse with the endothelial membrane and release S-nitrosothiols into the interstitial space between endothelial cells and muscle. L-amino acid transporters (L-AT) on the muscle cells then transport S-nitrosothiols that are also able to directly activate sGC resulting in vasodilation.

There remains the possibility that IR light at this wavelength has endothelial-specific effects, independent of the ability to generate heat. Coupling the observations that IR-induced relaxations were largely endothelium-dependent and blocked by ODQ certainly suggests that the primary mechanism of action underlying IR-induced relaxation of the occipital artery segments involves the actions of nitric oxide/S-nitrosothiols that elicit their effects via the activation of soluble guanylate cyclase and the generation of cGMP. Red/near IR light is reported to stimulate Ca^2+^ influx and Ca^2+^ release from the endoplasmic reticulum (Golovynska et al., 2020) degradation of preformed endothelial pools of S-nitrosothiols to nitric oxide (Keszler et al., 2017; Keszler et al., 2018; Keszler et al., 2019; Weihrauch et al., 2021; Keszler et al., 2022), and release of S-nitrosothiol-containing vesicles into the interstitial space (Riego et al., 2009). However, light in this wavelength has very low absorbance by water, meaning heat is not a likely mechanism. It is plausible that IR elicits endothelium-dependent vasodilation via 1) degradation of preformed S-nitrosothiols to nitric oxide which relaxes adjacent vascular smooth muscle via soluble-guanylate cyclase/cGMP-dependent mechanisms, and/or 2) release of preformed vesicular pools of S-nitrosothiols (Keszler et al., 2017; Keszler et al., 2018; Keszler et al., 2019; Keszler et al., 2022) or vesicle-containing S-nitrosothiols into the interstitial space between endothelial cells and muscle (Weihrauch et al., 2021) that are also able to directly activate soluble guanylate cyclase (Riego et al., 2009; Tsai and Hamblin, 2017). As depicted in Figure 9, suggests two potential mechanisms by which IR elicits vasodilation. As seen in panel **A**, IR may directly degrade preformed S-nitrosothiols to nitric oxide (NO) which diffuses across the membrane and relaxes adjacent vascular smooth muscle via soluble-guanylate cyclase (sGC)/cGMP-dependent mechanisms. As seen in panel **B**, IR may stimulate vesicles to fuse with the endothelial membrane and release S-nitrosothiols into the interstitial space between endothelial cells and muscle. L-amino acid transporters (L-AT) on the muscle cells then transport S-nitrosothiols that are also able to directly activate sGC to cause vasodilation. No matter what the mechanism, the longevity of the vasodilator response following termination of the short burst of IR is a surprising but welcome phenomenon in the context of developing a therapeutic strategy to improve vascular blood flow. Potential mechanisms could involve the burst of IR eliciting increases in intracellular mediators (e.g., Ca^2+^, cGMP, cAMP) and/or changes in phosphorylation/nitrosation status of functional proteins within endothelial cells (including mitochondria) that only slowly wane over time.

There are several limitations of our study, including the use of isolated blood vessels in a bath which removes the possible concomitant impact of IR on blood cells and surrounding tissues including active sympathetic nerve terminals which innervate blood vessels, including the cerebral (Nielsen and Owman, 1967) and peripheral (Aalkjær et al., 2021) vasculature. Although we could not detect temperature changes in the bath solution during/after bursts of IR, this does not preclude that IR increased the temperature of the occipital artery segments locally. Whether such an increase in temperature contributed to the greater endothelium-responsiveness of the proximal occipital artery segments remains to be established but the possibility that the expression of thermal- or light-sensitive proteins and/or pre-formed pools of S-nitrosothiols is higher in the proximal segments is currently under study. Also, in this study, we only report the effects of one energy. Each individual artery only received one application of IR light. There may be cumulative effects of multiple IR exposures, and therefore we did not demonstrate a range of energy effects. Other energies, or repeated IR exposures, would be important to investigate in the future.

The results of the present study may impact the interpretation of findings related to the effects and therapeutic benefits of infrared neuromodulation on intact tissues. It would be expected that an increase in blood flow within tissues would have a positive impact on tissue health. For example, IR modulation of neuronal function has the possibility of overcoming many of the issues that exist with more traditional electrical stimulation approaches such as the requirement for direct contact, lack of spatial resolution, and stimulation artifacts (Richter et al., 2011). Physiological responses elicited by brief pulses of IR light have been demonstrated in a variety of tissues, including the peripheral and central nervous systems (Chernov and Roe, 2014), and can both excite (Wells et al., 2005) and inhibit (Duke et al., 2013) neural activity. While stimulation of the vagus nerve using traditional electrical techniques has shown effects on conditions like hypertension (Plachta et al., 2014) and inflammation (Borovikova et al., 2000) among others, optical techniques offer more refined control of stimulation such as the ability to selectively inhibit small-diameter axons (Lothet et al., 2017) and thus provide more targeted modulation of specific physiological functions. Indeed, IR selectively depolarizes dorsal root ganglion and nodose ganglion neurons (Katz et al., 2010). As the nodose ganglion contains the majority of the cell bodies of vagal afferent neurons emanating from peripheral organs and structures (see Lewis et al., 1990; Lacolley et al., 2006b; Lacolley et al., 2006) application of IR to the cell bodies of autonomic and sensory nerves would be a unique manner to control visceral function including blood pressure, breathing, swallowing, airway resistance and pain perception. The results of the present study suggest that potential concomitant changes in blood flow in the above structures should be taken into consideration when interpreting the effects of IR and the therapeutic potential of IR protocols.

In conclusion, the present study suggests that IR generates rapid vasodilation in isolated occipital artery segments by mechanisms that may involve the release of nitric oxide from the endothelium that relaxes vascular smooth muscle by sGC-dependent processes. The use of light, and in particular, red light, has been explored for many clinical uses (see Tsai and Hamblin, 2017), including wound healing (Desmet et al., 2006), neuroprotective (Zhu et al., 2021), and tissue and bone regeneration (Wan et al., 2020). While the vasodilatory effects of red and near-IR take minutes to reach full effect (Keszler et al., 2017; Keszler et al., 2018; Keszler et al., 2019; Weihrauch et al., 2021; Keszler et al., 2022), we now demonstrate that direct application of IR elicits potent vasorelaxant effects in seconds that are maintained well beyond the period of IR exposure and that vessels maintain normal response to pharmacological stimuli after IR exposure. The application of IR may provide a new therapeutic option for increasing blood flow to targeted tissues in disease states such as hypertension and diabetes.

## Data Availability

The raw data supporting the conclusion of this article will be made available by the authors, without undue reservation.

## Abbreviations

5-HT, 5-hydroxytryptamine; AVP, arginine vasopressin; ECA, external carotid artery; HIK, high potassium PSS; IR, infrared light; ODQ, 1H-[1,2,4]oxadiazolo [4,3-a]quinoxalin-1-one; PSS, physiological saline solution.
